# Intelligent Algorithm-Based Picture Archiving and Communication System of MRI Images and Radiology Information System-Based Medical Informatization

**DOI:** 10.1155/2021/4997329

**Published:** 2021-09-17

**Authors:** Biao Liu, Baogao Tan, Lidi Huang, Jingxin Wei, Xulin Mo, Jintian Zheng, Hanchuan Luo

**Affiliations:** ^1^Department of Radiology, Guigang People's Hospital, Guigang 537100, Guangxi, China; ^2^Department of Hepatobiliary Pancreatic Surgery, Guigang People's Hospital, Guigang 537100, Guangxi, China

## Abstract

**Objective:**

The study aimed to explore the application value of picture archiving and communication system (PCAS) of MRI images based on radial basis function (RBF) neural network algorithm combined with the radiology information system (RIS).

**Methods:**

551 patients who required MRI examination in a hospital from May 2016 to May 2021 were selected as research subjects. Patients were divided into two groups according to their own wishes. Those who were willing to use the RBF neural network algorithm-based PCAS of MRI images combined with RIS were set as the combined group, involving a total of 278 cases; those who were unwilling were set as the regular group, involving a total of 273 cases. The RBF neural network algorithm-based PCAS of MRI images combined with RIS was trained and tested for classification performance and then used for comparison analysis.

**Result:**

The actual output (0.031259–0.038515) of all test samples was almost the same as the target output (0.000000) (*P* *>* 0.05). In the first 50,000 learnings, the iteration error of the RBF neural network dropped rapidly and finally stabilized at 0.038. The classification accuracy of the RBF neural network algorithm-based PCAS of MRI images combined with RIS for the head was 94.28%, that of abdomen was 97.22%, and it was 93.10% for knee joint, showing no statistically significant differences (*P* *>* 0.05), and the total classification accuracy was as high as 95%. The time spent in the examination in the combined group was about 2 hours, and that in the regular group was about 4 hours (*P* *>* 0.05). The satisfaction of the combined group (96.76%) was significantly higher than that of the control group (46.89%) (*P* *>* 0.05).

**Conclusion:**

The RBF neural network has good classification performance for MRI images. To incorporate intelligent algorithms into the medical information system can optimize the system. RBF has good application prospects in the medical information system, and it is worthy of continuous exploration.

## 1. Introduction

Medical informatization refers to the digitization and informatization of medical services [1]. As the name suggests, the patient's information is processed using the digital information technology. Medical informatization is a major trend worldwide in the medical field, especially in China [2, 3]. As a world-renowned country with a large population, and with the aging of the population, the demand for medical services is conceivable. Therefore, the accessibility, quality, and efficiency of medical services must be improved. With the advancement of computer technology and the continuous development of the National Golden Sanitation Project, many Internet-based medical information systems have been constructed, such as hospital information system (HIS), picture archiving and communication systems (PACS), and radiology information system (RIS) [4–6]. PACS and RIS are the focuses of this research.

The main task of PACS is to store various medical imaging data of patients digitally. When needed, they can be accessible under certain authorization, and some auxiliary diagnostic functions are incorporated at the same time [7–9]. The main function of RIS is to realize the networked control and management of medical imaging examination and sharing of medical graphics and text information and to realize remote treatment on this basis [10]. Compared with the traditional model, the digitalized PACS and RIS can raise the efficiency of treatment and reduce the waste of medical resources. To further improve these models, people have proposed intelligent algorithms, and the artificial neural network algorithm can optimize the computer model. Artificial neural network [11] is composed of components and physical processing units. It is used to simulate the structure and function of the biological neural network of human brain, trying to simplify, abstract, and simulate the biological neural network. Radial basis function (RBF) neural network [12, 13] is a feedforward neural network. It is characterized by the simple structure, fast training speed, strong function approximation ability, and classification ability. The system based on this network is bounded and stable. Above, the RBF neural network is widely used in various fields. Especially, it uses linear learning algorithms to solve the classification problems. Not only can the accuracy of the results be guaranteed but also the efficiency of the classification work can be lifted [14–16].

Therefore, in this study, the RBF neural network is used to optimize the PACS of MRI images, and then, a new medical information system is designed combined with the RIS. Next, its performance is analyzed, expected to provide more solutions to reduce the pressure in managing medical information.

## 2. Research Methods

### 2.1. MRI Examination

In this study, 551 patients admitted to the hospital from May 2016 to May 2021 who had the MRI examination were selected as research subjects, including 291 male patients and 260 female patients, aged between 22 and 56 years old, with an average age of 42.22 ± 9.13 years old. They were divided into two groups according to their own wishes. Those who were willing to use the RBF-based PCAS of MRI images combined with the RIS were defined as the combined group, with a total of 278 cases, and those who were unwilling were set as the regular group, with a total of 273 cases. This study has been approved by the Medical Ethics Committee.

Inclusion criteria: (a) patients aged 20–60 years old; (b) the patients had no mental disturbances; (c) patients with complete MRI data; (d) the patients and their families understood this study and had signed an informed consent form.

Exclusion criteria: (a) patients with severe visceral dysfunction and unconsciousness; (b) patients with blurred and incomplete MRI images.

### 2.2. MRI Image Classification Model Based on RBF Neural Network

#### 2.2.1. RBF Neural Network Model

The independent variable in the activation function of the RBF neural network is the distance *D* between the input and the weight vector, expressed as follows:

(1) $$\begin{array}{c} \left(D_{\text{RBF}}\right)=w^{-D^{2}}. \end{array}$$

**Figure 1:**
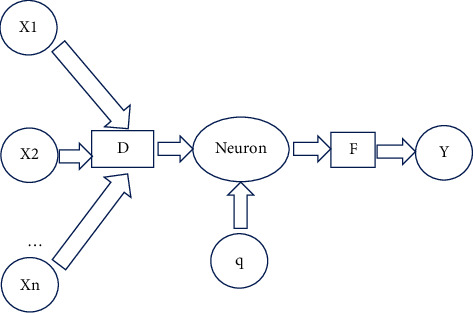
*D*_RBF_ model of RBF algorithm.

According to equation (1), when *D* decreases, the output increases. When *D* is 0, that is to say, when the input vector coincides with the weight vector, the output value *D*_RBF_ = 1. The RBF neural network model is shown in Figure 1, where *q* is the threshold for adjusting the sensitivity of the neuron.

**Figure 2:**
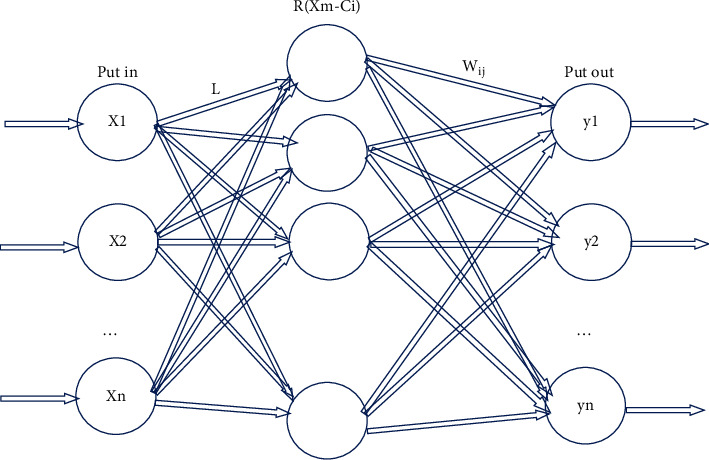
The structure of the RBF neural network.

The RBF neural network is composed of the input layer, the hidden layer, and the output layer. It is a three-layer network, and the specific network structure is shown in Figure 2.

In Figure 2, *X*=(*x*_1_, *x*_2_, ...*x*_*n*_) refers to the input vector. The input layer only transmits signals, and *L* is the weight of the connection between the input layer and the hidden layer. The hidden layer is RBF, a Gaussian function, expressed as follows:

(2) $$\begin{array}{c} \text{RBF}\left(X_{e}-c_{i}\right)\exp\left(-\frac{L}{2\sigma_{i}^{2}}{\left\|X_{e}-c_{i}\right\|}^{2}\right), \end{array}$$

where ‖*X*_*e*_ − *c*_*i*_‖ represents the European norm, *X*_*e*_=(*x*_1_^*e*^, *x*_2_^*e*^, ...*x*_*m*_^*e*^) represents the *e*-th input sample *e* = 1,2, ..., *E*, *E* refers to the total number of data samples, *i* = 1,2, ..., *g* represents the number of nodes in the hidden layer, *c*_*i*_ represents the center of the Gaussian function, *σ*_*i*_ represents the variance of the Gaussian function, expressed as follows:

(3) $$\begin{array}{c} \sigma_{i}=\frac{c_{\max}}{\sqrt{2g}}, \end{array}$$

where *c*_max_ represents the maximum distance between the selected centers. According to Figure 2, the network output is expressed as follows:

(4) $$\begin{array}{c} y_{i}=\sum_{i=1}^{h}\omega_{ij}R\left(X_{e}-c_{i}\right),\\ \sum_{j=0}^{n}y_{i}=1, \end{array}$$

where *j* = 1, 2, ..., *n* represents the number of nodes in the output layer, *y*_*i*_ ∈ (0,1) represents the actual output of the node corresponding to the input sample, which also represents the probability of belonging to class *j*. *Y*=(*y*_1_, *y*_2_, ...*y*_*n*_) is the output vector, and *ω*_*ij*_ is the connection weight between the output layer and the hidden layer, which can be directly calculated by the least square method.

(5) $$\begin{array}{c} \omega_{ij}=\exp\left(\frac{h}{c_{\max}}{\left\|X_{e}-c_{i}\right\|}^{2}\right). \end{array}$$

The output of the RBF neural network is the linear weighted sum of the output of each node in the hidden layer [8, 9]. The weight between the hidden layer and the output layer is adjustable. To determine whether the weights need to be adjusted, the following error equation is used.

(6) $$\begin{array}{c} U=\frac{\sum_{e=1}^{e}{\left(Y_{e}-D_{e}\right)}^{2}}{2*E}, \end{array}$$

where *Y*_*e*_=(*y*_1_^*e*^, *y*_2_^*e*^, ...*y*_*n*_^*e*^) and *D*_*e*_=(*d*_1_^*e*^, *d*_2_^*e*^, ...*d*_*n*_^*e*^) are the actual output and ideal output of the *e*-th training sample, respectively.

#### 2.2.2. Training Steps of RBF Neural Network

 Step 1: the extracted local feature vector (see the following equation, *e* = 1, 2,…, *E*) constitutes the training set Ω=(*X*_1_, *X*_2_,…, *X*_*e*_) of the neural network.
  (7) $$\begin{array}{c} X^{e}=\left(x^{e},y^{e},f^{e},{\left\|\nabla f\right\|}^{e},d_{1}^{e},\ldots ,d_{j}^{e},\ldots d_{n}^{e}\right). \end{array}$$
 Step 2: the number of hidden layer nodes is set to *k*, and *k* training samples are selected as the clustering centers *c*_*i*_(*I*), corresponding to the set Γ_*i*_, where *i* = 1, 2, ..., *k* and the number of iteration operations *I* = 1.
 Step 3: the Euclidean distance *D*(*X*_*e*_, *c*_*i*_(*I*)) between the training sample *X*_*e*_ and the center *c*_*i*_ is calculated as follows:
  (8) $$\begin{array}{c} D\left(X_{e},c_{i}\left(I\right)\right)=\min\left\{D\left(X_{e},c_{i}\left(I\right)\right),\;i=1,2,...,k\right\}. \end{array}$$
 Then,
  (9) $$\begin{array}{c} X_{e}\in \Gamma_{i}. \end{array}$$
 Step 4: the cluster center of *Γ i* is recalculated:
  (10) $$\begin{array}{c} c_{i}\left(I+1\right)=\frac{1}{M_{i}}\sum_{e=1}^{M_{i}}X_{e}^{\left(i\right)}, \end{array}$$
 where *M*_*i*_ represents the number of Γ_*i*_ sample *X*_*e*_^(*i*)^.
 If
  (11) $$\begin{array}{c} c_{i}\left(I+1\right)\neq c_{i}\left(I\right), \end{array}$$
 then
  (12) $$\begin{array}{c} I=I+1. \end{array}$$
 Return to step 3; otherwise, the final cluster center is *c*_*i*_.
 Step 5: the variance *σ*_*i*_ of the Gaussian function is calculated as follows:
  (13) $$\begin{array}{c} \sigma_{i}=\frac{c_{\max}}{\sqrt{2g}}, \end{array}$$
 where *i* = 1,2, ...; *g* represents the number of nodes in the hidden layer; and *c*_max_ represents the maximum distance between the selected centers.
 Step 6: the weight *ω*_*ij*_ between the hidden layer and the output layer and the actual network output *Y*_*p*_ are calculated:
  (14) $$\begin{array}{c} y_{i}=\sum_{i=1}^{h}\omega_{ij}R\left(X_{e}-c_{i}\right), \end{array}$$
  (15) $$\begin{array}{c} \omega_{ij}=\exp\left(\frac{h}{c_{\max}}{\left\|X_{e}-c_{i}\right\|}^{2}\right). \end{array}$$
 Step 7: equation (16) is used to judge whether the training termination condition is met. If it is satisfied, the neural network training is finished; otherwise, return to step (15).

(16) $$\begin{array}{c} P=\frac{\sum_{e=1}^{e}{\left(Y_{e}-D_{e}\right)}^{2}}{2*E}. \end{array}$$

Finally, based on the classification results of the neural network, different RGB values are assigned to different types of voxels to classify medical images. A few pixels are extracted to construct a test sample. At this time, the pixels extracted in step (1) should be excluded. The test samples are input into the network obtained by equation (17), and the classification performance of the RBF neural network is judged according to the output:

(17) $$\begin{array}{c} \sigma_{i}=\frac{c_{\max}}{\sqrt{2g}}. \end{array}$$

### 2.3. MRI Detection Method

All patients were examined by the same radiology operator for MRI, and reasonable examination methods and appropriate scanning parameters were used according to the different parts of the body. The MRI images of the 217 patients in the combined group were classified using the RBF neural network algorithm and then archived and imported into the RIS.

### 2.4. Test Index

**Figure 3:**
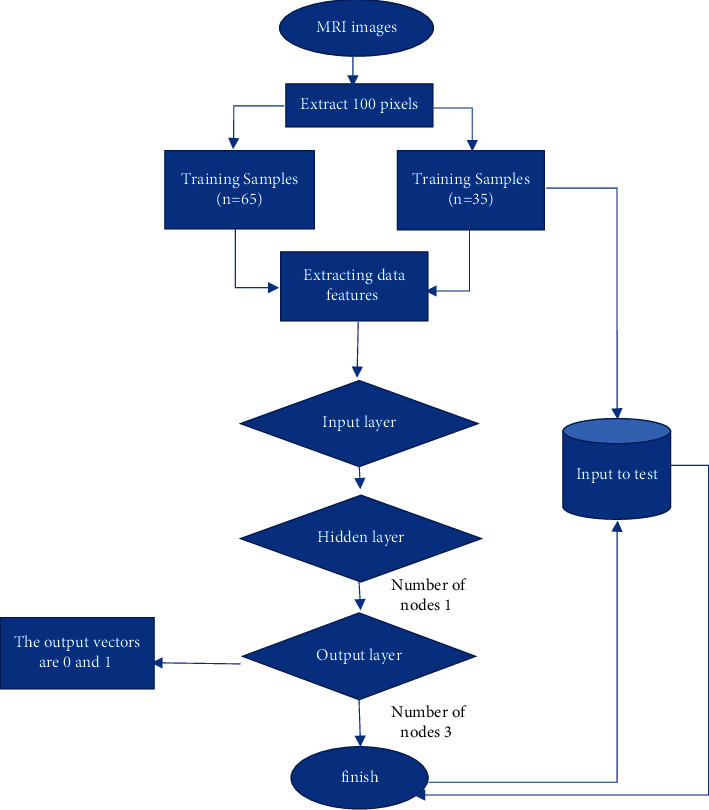
Classification flowchart of the RBF neural network.

First, the classification performance of the RBF neural network is detected. The detection method is shown in Figure 3, and the classification accuracy is then identified. Then, if demonstrating good performance, it will be used in clinical experimental research; if the performance is poor, it will continue to be optimized. Next, according to the time required for the examination, the efficiency of the RBF-based PCAS of MRI images combined with the RIS was analyzed, and the satisfaction rates in the combined group and the regular group were then investigated by face-to-face consultation.

### 2.5. Statistical Methods

SPSS22.0 statistical analysis software was used to process the data, the measurement data were expressed as mean ± standard deviation, and the data comparison adopted analysis of variance. *P* *<* 0.05 was the threshold for significance.

## 3. Results

### 3.1. Classification Performance of RBF Neural Network

**Figure 4:**
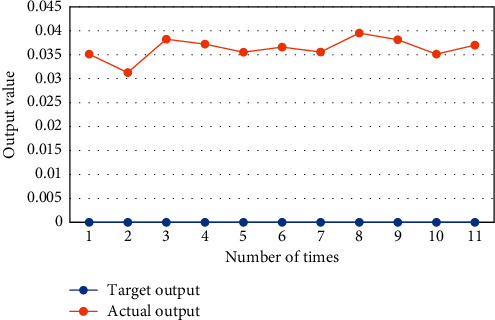
Output results of test samples.

Figure 4 showed the test results of the classification performance of the RBF neural network. It was noted that the actual output (0.000 000) of all test samples was almost the same as the target output (0.031259–0.038515), indicating that the network can effectively distinguish this type of data. Experiments showed that the RBF neural network demonstrated superb capabilities in image classification, and it should be suggested in clinic.

### 3.2. MRI Image Segmentation Effects

**Figure 5:**
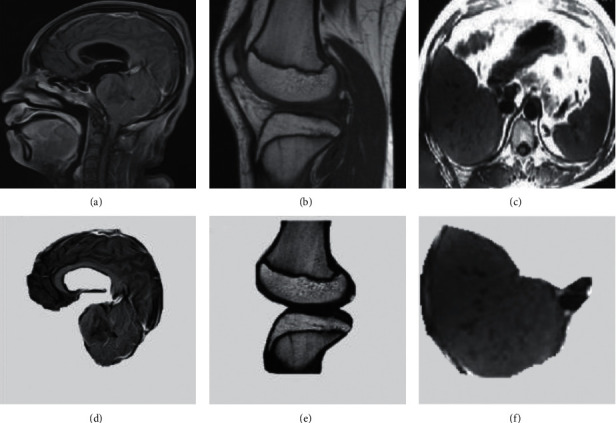
Segmentation results of MRI images of different parts of the body. (a–c) Head, abdomen, and knee joints are classified. (d–f) Brain, liver, and knee joint bones.

Figure 5 showed segmentation effects of MRI images of different parts. Figures 5(a)–5(c) were the original images of the brain, abdomen, and knee joints. According to the actual needs, the main observation parts were the brain, liver, and the knee joint space. Figures 5(d)–5(f) were the segmented results. It was noted that the brain, abdomen, and knee joints presented high signal pixels, while the other parts were white, showing good contrast.

### 3.3. Classification Effects

Table 1 shows the classification results of the RBF-based PCAS of MRI images combined with the RIS, with the actual lesions of 100 patients as the reference. It was noted that its classification accuracy of classification of head was 94.28%, that of abdomen was 97.22%, and it was 93.10% for the knee joint, showing no statistically significant differences (*P* *>* 0.05), and the total classification accuracy was as high as 95%.

### 3.4. Error in MRI Image Classification

Table 2 shows the iterative errors of the RBF neural network in the training process of image classification. In the first 50,000 learnings, the iterative error of the RBF neural network dropped rapidly and finally stabilized at about 0.038. Experiments showed that the medical image classification method based on the RBF neural network was characterized by stable convergence and fast speed while ensuring the correctness of classification.

### 3.5. Comparison of General Information of the Two Groups of Patients

#### 3.5.1. Gender Distribution

**Figure 6:**
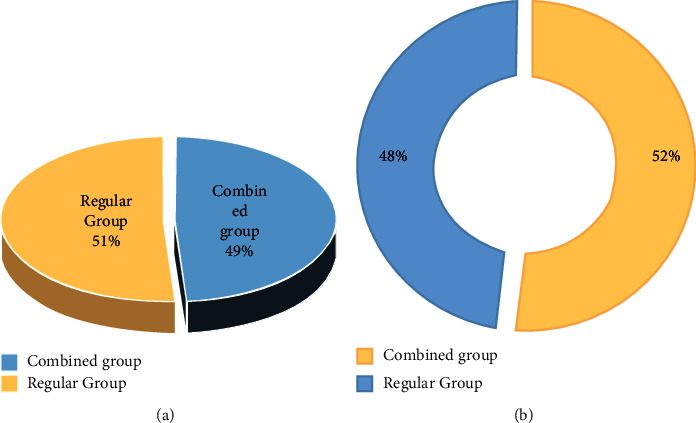
Gender distribution. (a) Female. (b) Male.

Figure 6 shows the gender distribution of the subjects in the combined group and the regular group.  Figure 6(a) shows the distribution of 260 females in the two groups. In the combined group, there were 127 females, accounting for 48.85%; while in the regular group, there were 133 females, accounting for 51.15%.  Figure 6(b) shows the distribution of 291 males in the combined group and the regular group. In the combined group, there were 151 males, accounting for 51.89%, and in the regular group, there were 140 males, accounting for 48.11%. After comparison, there was no significant difference in the distribution of males and females between the combined group and the regular group (*P* *>* 0.05).

#### 3.5.2. Age Distribution

We divided the ages into 21–30, 31–40, 41–50, and above 50.  Table 3 shows the distribution of the ages of the combined group and the regular group. It was noted that the number of people aged between 41 and 50 and 50 years old and above accounted for 79.85% and 80.22% of the total number, respectively, in the combined group and the regular group, and there was no statistically significant difference (*P* *>* 0.05).

#### 3.5.3. Distribution of Parts of the Body Requiring MRI

**Figure 7:**
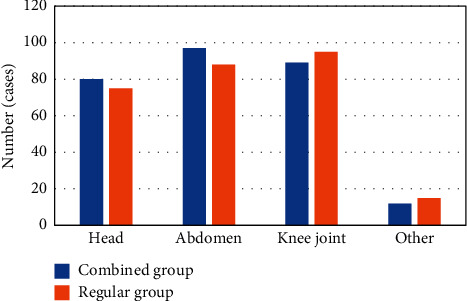
The distribution of parts of the body requiring MRI.

In this study, the MRI examination was mainly performed on the head, abdomen, and knee joints, while other parts accounted for a very small number. Of the 278 patients in the combined group, 80 required head MRI, 97 required abdominal MRI, 89 required knee MRI, and 12 required MRI for other parts. Of the 273 patients in the regular group, 75 had head MRI examination, 88 had abdominal examination, 95 had knee joint examination, and 15 had MRI for other parts. After comparison, there was no significant difference in the number of each part of the two groups (*P* *>* 0.05), as shown in Figure 7.

### 3.6. Time-Consuming and Satisfaction of the Two Groups of Patients

**Figure 8:**
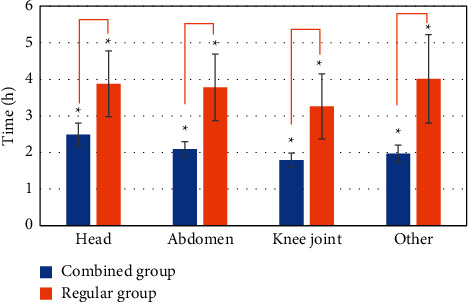
Time spent for MRI examination.

**Figure 9:**
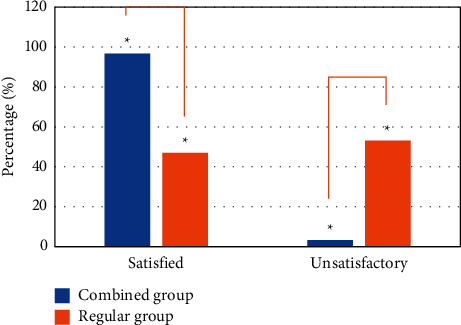
Satisfaction survey results.

Figure 8 shows the time spent in the examination of different parts of patients in the combined group and the regular group. It was noted that the time required for the examination in the combined group was basically about 2 hours, while the time in the regular group was more than 3 hours and close to 4 hours, and there was a statistically significant difference between the two groups (*P* *<* 0.05).  Figure 9 showed whether the two groups of patients were satisfied with the time required for the examination. In the combined group, 269 subjects were satisfied, accounting for 96.76%, while in the regular group, the number was 128, accounting for 46.89%. The dissatisfaction rate was 3.24% and 53.11%, respectively. The satisfaction rate of patients in the combined group was higher than that of the control group, and the dissatisfaction rate was lower than that of the control group (*P* *<* 0.05).

## 4. Discussion

This study focused on the clinical application of PCAS of MRI image based on the RBF neural network algorithm combined with RIS. In terms of classification effects of RBF, the results showed that the RBF neural network demonstrated superb capabilities in classification of MRI images, and it should be suggested in clinics to classify MRI images. An experiment using the RBF neural network to classify MRI images showed that the structure of the RBF neural network was simple, the number of learning weights was small, and the convergence speed was fast. It can effectively distinguish different structures in medical images and display image details. At the same time, the error curve had stable convergence [12]. The classification accuracy of the RBF-based PCAS of MRI images combined with the RIS for head was 94.28%, that of the abdomen was 97.22%, and it was 93.10% for the knee joint, showing no statistically significant differences (*P* *>* 0.05), and the overall classification accuracy was as high as 95%, consistent with the results of most studies. Karakitsos et al. [17] once discussed the potential of morphometry and neural network tools in identifying benign and malignant cell nuclei and lesions in the lower urinary tract. They found that the RBF classifier correctly classified 93.64% of benign cell nuclei and 85.61% of malignant cell nuclei, and the overall accuracy rate was 84.45%. At the patient level, the overall accuracy of the RBF classifier was 94.97%. Behr et al. [18] carried out a case-control experiment to study the feasibility of a support vector machine classifier for myofascial pain syndrome. They found that the accuracy of the RBF classifier was 86.96, the Matthews correlation coefficient was 0.724, the sensitivity was 88%, and the specificity was 86%. Both showed good classification effects of RBF, which was basically consistent with the results of this study.

Then, RIS was combined to form a new medical information system for clinical research analysis. The results showed that the total time required was reduced by about half compared to that in the regular group, and 96.76% of the people in the combined group were satisfied with it, while in the regular group, the satisfaction rate was 46.89%, and the difference was statistically significant between the two groups (*P* *<* 0.05), indicating that incorporating intelligent algorithms into medical information system optimized the original system. A research on a smart medical handheld mobile nursing information system based on intelligent algorithms found that, by setting up an information retrieval module and an information modification module, it is possible to quickly and effectively assign relevant nursing information to medical staff and strengthen nurses' understanding of relevant information [19]. There are also a large number of studies on intelligent algorithms and medical information systems indicating that intelligent algorithms can enhance the functions and efficiency of medical information systems [20–22]. The comparative analysis revealed a high degree of consistency between research conclusions of other experts with the results of this research to support the application of intelligent algorithms in medical information systems.

## 5. Conclusion

The research focused on the application of PCAS of MRI image based on the RBF neural network algorithm combined with RIS. The results showed that the RBF neural network has good classification performance for MRI images and can effectively classify the images, indicating incorporating intelligent algorithms into medical information system can optimize the system. However, due to the single source of the research samples, the research results lack representativeness, which should be improved in the subsequent research. In conclusion, RBF has good application prospects in medical information systems, and it is worthy of continuous exploration.

## Figures and Tables

**Table 1:** Classification result statistics.

	Head	Abdomen	Knee joint	Total
Classification results (*n* = 100 cases)	33	35	27	95
Actual results (*n* = 100 cases)	35	36	29	100
Accuracy (%)	94.28	97.22	93.10	95.00

**Table 2:** The error in MRI image classification.

Times of training	0	10 × 10^4^	20 × 10^4^	30 × 10^4^	40 × 10^4^	50 × 10^4^	60 × 10^4^	70 × 10^4^	80 × 10^4^	90 × 10^4^
Iteration error	0.175	0.045	0.044	0.042	0.041	0.041	0.038	0.038	0.038	0.038

**Table 3:** Age distribution of the subjects.

Age (year)	21–30	31–40	41–50	Above 50
Grouping				
Combined group (*n* = 278)	23 (8.27%)	33 (11.87%)	167 (60.07%)	55 (19.78%)
Regular group (*n* = 273)	30 (10.99%)	24 (8.79%)	178 (65.20%)	41 (15.02%)

## Data Availability

The data used to support the findings of this study are available from the corresponding author upon request.
